# Primary heart involvement assessed by cardiac magnetic resonance is associated with capillaroscopy abnormalities in systemic sclerosis

**DOI:** 10.1186/s42358-026-00549-9

**Published:** 2026-06-19

**Authors:** Lucas Martins, Marly Uellendahl, Gabriel Ferreira, Cristiane Kayser

**Affiliations:** 1 Rheumatology Division, Escola Paulista de Medicina, Universidade Federal de São Paulo, Rua dos Otonis 863, 2º andar, Vila Clementino, São Paulo, SP 04025-002 Brasil; 2 Cardiology Division, Escola Paulista de Medicina, Universidade Federal de São Paulo, São Paulo, SP Brasil; 3 Diagnósticos da América S.A. (DASA), São Paulo, SP Brasil

**Keywords:** Systemic sclerosis, Heart involvement, Cardiac magnetic resonance imaging, Nailfold capillaroscopy

## Abstract

**Background:**

Heart involvement is a common complication in patients with systemic sclerosis (SSc). Primary heart involvement associated with SSc (SSc-HI) can be assessed via cardiac magnetic resonance (CMR), a sensitive method that evaluates morphology, function, and tissue characteristics indicative of microvascular dysfunction, inflammatory, and fibrotic changes. This study aimed to investigate the relationship between SSc-HI evaluated by CMR after cold stress protocol and the peripheral vasculopathy assessed by nailfold capillaroscopy in SSc patients.

**Methods:**

A cross-sectional exploratory study including 40 SSc patients was performed. CMR measured morphology, function, and strain. SSc-HI CMR vasculopathy-related lesions were evaluated by perfusion defects and myocardial perfusion reserve (MPR); fibrosis-related lesions were evaluated by late gadolinium enhancement (LGE), reduced left ventricular global longitudinal strain (LV-GLS), and biventricular failure. The following parameters were evaluated via videocapillaroscopy: mean capillary/mm, giant capillaries, avascular score, and the presence of a late scleroderma (SD) or non-late SD pattern.

**Results:**

SSc-HI on CMR was observed in 22 (55%) patients. Vasculopathy and fibrosis-related lesions were observed in 15 (37.5%) and 8 (20%) patients, respectively. The late SD pattern group had a significantly higher frequency of moderate/severe pericardial effusion (*p* = 0.04) and a higher frequency of fibrosis-related lesions (*p* = 0.01), with more patients presenting with an LV-GLS < 14% (*p* = 0.01) and LGE (*p* < 0.01) than in the patients with the non-late SD pattern. A lower number of capillary loops/mm and a higher avascular score were associated with fibrosis-related lesions (*p* < 0.01 and *p* = 0.01, respectively), myocardial involvement (*p* < 0.01, both) and any SSc-HI (*p* = 0.01 and *p* < 0.01, respectively).

**Conclusions:**

SSc-HI CMR changes indicative of vasculopathy and fibrosis were frequently observed and associated with more severe capillaroscopic abnormalities, suggesting that the peripheral microcirculation may mirror cardiac injuries. Thus, the association between CMR and capillaroscopy indicated that capillaroscopy changes might be associated with SSc-HI.

**Supplementary Information:**

The online version contains supplementary material available at https://doi.org/10.1186/s42358-026-00549-9.

## Background

Systemic sclerosis (SSc) is an immune-mediated rheumatic disease characterized by a distinct triad of vasculopathy, autoimmunity, and fibrosis of the skin and visceral organs [1, 2]. Primary heart involvement caused by SSc (SSc-HI) is an important manifestation and a frequent cause of death in SSc patients [3]. The prevalence of SSc-HI varies from 7 to 44%, depending on the definition of primary heart involvement, and can reach up to 90% in autopsy studies. SSc-HI is often clinically occult, and when it manifests, it presents with heart failure, arrhythmia, or pericardial involvement and is associated with a poor prognosis [4, 5].

Like SSc, SSc-HI pathogenesis involves three mechanisms: vasculopathy (microvascular abnormalities), inflammation, and fibrosis [6–8]. Microvascular involvement is associated with endothelial damage and abnormal vasoreactivity, which may lead to ischaemia‒reperfusion cycles in the cardiac microcirculation, akin to a cardiac Raynaud’s phenomenon [9–12]. In addition, immune-mediated inflammatory involvement of the myocardium or pericardium has been described in SSc-HI patients [13, 14]. Finally, microvascular and inflammatory abnormalities may lead to myocardial fibrosis, which is characterized by biventricular, subendocardial, and noncoronary distribution [15–17]. Histopathological evaluation of SSc-HI revealed a pattern of plaque-like fibrosis with focal myocardial necrosis of the contraction band [18, 19].

Compared with several methods that can be used to assess cardiac involvement in SSc, cardiac magnetic resonance (CMR) is currently considered the gold standard in the assessment of cardiac volume and ejection fraction [20]. Moreover, CMR has greater sensitivity than echocardiography for detecting early cardiac changes in SSc-HI [21, 22]. Different imaging protocols in CMR allow the assessment of lesions indicative of microvascular, inflammatory, and fibrotic changes [23]. Abnormalities related to SSc-HI vasculopathy have been documented by the presence of perfusion defects [24, 25] or by changes in quantitative perfusion after pharmacological or cold stress, such as lower vasodilatory capacity in SSc patients after cold stress [12, 26]. Myocardial inflammation and fibrosis have also been demonstrated in different studies by the presence of myocardial oedema, late gadolinium enhancement (LGE) imaging, or parametric T1 or T2 mapping images with diffuse inflammation or fibrosis [13, 16, 27–29].

Nailfold capillaroscopy is a non-invasive method utilized for evaluating structural changes in the peripheral microcirculation [30]. Patients with SSc typically present abnormalities such as capillary dilation, reduced capillary density, avascular areas, microhemorrhages, and disorganization of the nailfold capillary architecture, which is known as the scleroderma (SD) pattern. The SD pattern is categorized into three subtypes: early, active, and late patterns, according to the degree of capillary dilation and capillary loss [31]. The late SD pattern and more severe capillary abnormalities are associated with more advanced disease and the involvement of certain organs [32–35]. Regarding heart involvement, two studies have shown an association of the late SD pattern with cardiac abnormalities evaluated by electrocardiogram or echocardiography and/or elevated NT-pro-BNP levels [34, 36]. However, the role of nailfold capillaroscopy in the context of CMR evaluation of SSc-HI is unknown.

The aim of this study was to investigate the association between cardiac involvement evaluated by CMR, including assessment of myocardial perfusion before and after cold stress, and the peripheral vasculopathy evaluated by nailfold capillaroscopy in SSc patients.

## Methods

### Study design and patient population

We performed a cross-sectional single-centre exploratory study that included 40 SSc patients consecutively recruited between December 2021 and January 2024. All participants underwent CMR and nailfold capillaroscopy within 4 weeks of each medical procedure. All participants signed informed consent, and the study was approved by the local ethics committee (study number: 2.468.573).

All SSc patients fulfilled the American College of Rheumatology/European League Against Rheumatism 2013 classification criteria for SSc [37]. Exclusion criteria included smoking, pregnancy, previous cardiovascular disease (coronary artery disease, myocardial infarct, stroke, severe arrhythmia, or heart failure), pulmonary arterial hypertension (PAH) diagnosed with right heart catheterization, diabetes mellitus, BMI > 35 kg/m², and contraindications for performing CMR (Glomerular filtration rate < 50 mL/min, implanted medical device, or claustrophobia). Patients with controlled systemic arterial hypertension or dyslipidaemia were included. SSc clinical features, specific antibodies, and previous and current treatments with vasodilators or immunosuppressants were also recorded.

### Cardiac magnetic resonance protocol and post-processing

CMR was performed on a 1.5T MRI scanner (Avanto, Siemens, Germany). All patients discontinued vasodilators 72 h prior to the CMR. CINE-MRI data were acquired for the long and short cardiac axes. Perfusion imaging consisted of 2 sequences for evaluating left ventricular (LV) perfusion along the short axis: an arterial input fraction (AIF) sequence for calibration followed by the LV myocardial perfusion sequence. Perfusion imaging was performed at rest and was repeated after cold stress. Cold stress consisted of immersing the upper limb, without venipuncture, in a container of water at a temperature of 10° ± 2 °C for 3 min, adapted from Galea et al. [12]. Late gadolinium enhancement (LGE) imaging was performed 5 min after the cold stress perfusion sequence. Gadovist (Schering, Berlin, Germany) was the contrast agent injected for perfusion and LGE.

All acquired images were processed using the CVI42 software (Circle Cardiovascular Imaging, Calgary, Canada). The American Heart Association (AHA) cardiac segmentation model with 17 cardiac segments was used for segmental analysis of strain and quantitative perfusion images [38]. The CMR images were analyzed by two experienced evaluators blinded to the patients’ previous medical history.

CINE-MRI images were used to measure biventricular volumes (end-diastolic volume, end-systolic volume, and stroke volume), myocardial mass indexed by body surface area, and LV and right ventricular (RV) ejection fraction, and to evaluate pericarditis and pericardial effusion. Pericardial effusion was measured and categorized as laminar/mild (< 10 mm) or moderate/severe (> 10 mm) [39]. Additionally, the LV and RV global longitudinal strains (LV-GLS and RV-GLS) were calculated from CINE-MRI using the feature tracking method.

Volumetric and functional parameters were used to classify patients into five distinct CMR phenotypes: normal function with an average cavity, normal function with a small cavity, normal function with a large cavity, right ventricular failure, and biventricular failure, according to Knight et al. [29].

For perfusion imaging, cold-induced perfusion defects (PD) were visually assessed. Quantitative perfusion was determined by measuring myocardial blood flow (MBF) at rest and after cold stress. The myocardial perfusion reserve (MPR) was calculated by the ratio of the MBF after cold stress by the MBF at rest. An MPR < 1.0 was used to represent no vasodilatory response after cold stress. The delta MBF (percentage of change in MBF after cold stress) was also calculated, and a cut-off of a MBF < 33.8% was used as previously described [12]. The presence of LGE lesions was assessed.

Additional data on the CMR protocol, image acquisition, and post-processing are described in Supplementary Data S1 and Table S1.

Furthermore, SSc CMR findings were categorized into vasculopathy and fibrosis-related involvement. Cold-induced PD and an MPR < 1.0 were considered indicative of vasculopathy-related lesions. LGE lesions, a LV-GLS < 14% and biventricular failure were considered indicative of fibrosis-related lesions [29, 40–42].

### Nailfold videocapillaroscopy

Nailfold capillaroscopy was performed with a videocapillaroscope (NVC) (Videocap 8.14, DS-Medica, Italy) under 200x magnification. In all fingers, except for the thumbs, two consecutive images of at least 1 mm of the nailfold capillary network were captured. Each image was processed on a 1 mm grid in which the number of capillary loops per mm was counted. Capillary loops with an apical diameter greater than 50 μm were considered giant capillaries. An avascular score was subsequently applied to each image according to the number of capillary loops/mm (score 0: ≥ 9 capillary loops/mm; score 1: 7–8 capillary loops/mm; score 2: 4–6 capillary loops/mm; score 3: ≤ 3 capillary loops/mm). For each parameter, the mean number of 16 images was calculated. Furthermore, all NVCs findings were classified as normal, non-specific abnormalities, or SD patterns. The SD pattern was subclassified into early, active, or late SD patterns [31]. Finally, patients were categorized into late SD patterns or non-late SD patterns [43, 44].

### Statistical analysis

Statistical analyses were performed using Jamovi software version 2.2. The data are presented as the means with their standard deviations, medians and interquartile ranges, or frequencies and percentages. The Shapiro‒Wilk test was used to assess the normality of the distribution of continuous variables. Student’s t test and the Mann‒Whitney U test were used to compare normally distributed and non-normally distributed continuous variables, respectively. Fisher’s exact test was used to compare categorical variables. A Spearman’s test was performed to assess the correlation between the nailfold capillaroscopy and the CMR data. The Wilcoxon test was used to compare paired MBF values before and after cold stress.A P value of < 0.05 was considered statistically significant.

## Results

The mean age of the 40 SSc patients was 51 ± 12 years, the majority were female (95%) with diffuse cutaneous SSc (52.5%), and a mean disease duration of 7.5 ± 7.5 years. The most prevalent capillaroscopic pattern was the SD pattern, which was observed in 33 (82.5%) patients, 25% of those had a late SD pattern (Table 1).

**Table 1:** SSc patients’ baseline characteristics and videocapillaroscopy parameters

Variables	SSc (*n* = 40)
Age (years, mean ± SD)	51.0 ± 12.0
Female / male (n, %)	38 (95%)/2 (5%)
Diffuse / limited (n, %)	21 (52.5%)/19 (47.5%)
Disease duration (median ± IQR)	7.5 ± 7.2
Modified Rodnan skin score (median ± IQR)	5.5 ± 13.5
Friction rubs (n, %)	5 (12.5%)
Digital ulcers (n, %)	24 (60%)
Arthritis (n, %)	20 (50%)
Myositis (n, %)	5 (12.5%)
Interstitial lung disease (n, %)	22 (55%)
GI tract involvement (n, %)	36 (90%)
Scleroderma renal crisis (n, %)	1 (2.5%)
Anti-SCL-70 (n, %)	12 (30%)
Anti-centromere (n, %)	4 (10%)
Anti-RNA polymerase III (n, %)	1 (2.5%)
Treatment	
Calcium channel blockers (n, %)	39 (97.5%)
Phosphodiesterase-5 enzyme inhibitor (n, %)	19 (47.5%)
Endothelin receptor antagonist (n, %)	2 (5%)
Immunosuppressors (n, %)	33 (82.5%)
Videocapillaroscopy	
Normal pattern	1 (2.5%)
Non-specific abnormalities	6 (15%)
Scleroderma pattern (n, %)	33 (82.5%)
Early scleroderma pattern (n, %)	5 (12.5%)
Active scleroderma pattern (n, %)	18 (45%)
Late scleroderma pattern (n, %)	10 (25%)
Capillary loops/mm (mean ± SD)	5.78 ± 3.95
Avascular score (mean ± SD)	1.28 ± 1.90
Giant capillary (mean ± SD)	0.03 ± 0.82

All SSc patients underwent cold stress CMR, after which Raynaud’s phenomenon was presented in all patients; within two weeks of follow-up, none of the patients developed digital ulcers. In the post-processing analysis of CMR, the quantitative perfusion protocol was not adequately performed in 6 patients. Thus, the perfusion protocol was analysed in 34 patients. There was no difference between the MBF values at rest and after cold stress (*p* = 0.13). However, a delta MBF < 33.8% was found in 65% of the patients (Table 2).

**Table 2:** CMR in SSc patients according to the presence of late SD and non-late SD patterns via videocapillaroscopy

CMR parameters	SSc (*n* = 40)	Late SD (*n* = 10)	Non-late SD (*n* = 30)	*p* value
LV and RV morphology and function (mean ± SD)				
LV Indexed end diastolic volume (ml)	69.7 ± 14.6	75.5 ± 17.7	67.7 ± 13.1	0.13
LV Indexed end systolic volume (ml)	24.0 ± 11.5	28.5 ± 14.9	24.1 ± 6.7	0.69
LV Indexed stroke volume (ml)	44.4 ± 7.7	46.8 ± 7.6	43.5 ± 7.7	0.25
Indexed LV mass (g)	51.7 ± 11.2	60.7 ± 11.0	48.7 ± 9.7	< 0.01
LV ejection fraction (%)	63.5 ± 7.0	63.4 ± 10.6	64.4 ± 4.5	0.88
RV Indexed end diastolic volume (ml)	63.5 ± 17.5	74.0 ± 18.5	67.2 ± 15.1	0.24
RV Indexed end systolic volume (ml)	25.5 ± 7.7	29.1 ± 12.2	26.6 ± 8.6	0.63
RV Indexed stroke volume (ml)	41.6 ± 7.5	44.8 ± 6.9	40.6 ± 7.5	0.10
RV ejection fraction (%)	61.5 ± 5.4	61.7 ± 6.9	61.4 ± 5.0	1.00
Strain				
LV-GLS (mean ± SD)	16 ± 2%	14 ± 3%	17 ± 2%	< 0.01
LV-GLS < 14% (n, %)	5 (12.5%)	4 (40%)	1 (3.3%)	0.01
RV-GLS (%)	24 ± 4%	22 ± 4%	25 ± 4%	0.17
Quantitative perfusion (mean ± SD)	*n* = 34	*n* = 8	*n* = 26	
Cold-induced perfusion defects (n, %)	2 (5%)	0 (0%)	2 (6.7%)	1.00
MBF rest (ml/g/min)	1.5 ± 0.5	1.3 ± 0.3	1.5 ± 0.6	0.62
MBF stress (ml/g/min)	1.6 ± 0.6	1.6 ± 0.6	1.6 ± 0.6	0.42
MPR	1.1 ± 0.3	1.1 ± 0.2	1.1 ± 0.3	0.58
MPR < 1.0 (n, %)	13 (38.2%)	3 (37.5%)	10 (38.5%)	1.00
Delta MBF < 33.8% (n, %)	26 (65%)	6 (75%)	20 (76.9%)	1.00
Myocardial fibrosis				
LGE (n, %)	4 (10%)	4 (40%)	0 (0%)	< 0.01
Pericardial evaluation				
Laminar/small pericardial effusion (n, %)	12 (30%)	2 (20%)	10 (33.3%)	0.69
Moderate/severe pericardial effusion (n, %)	4 (10%)	3 (30%)	1 (3.3%)	0.04
Volumetric and functional classification				
Normal function and small cavity (n, %)	22 (55%)	4 (40%)	18 (60%)	0.30
Normal function and average cavity (n, %)	13 (32.5%)	3 (30%)	10 (33.3%)	1.00
Normal function and large cavity (n, %)	1 (2.5%)	1 (10%)	0 (0%)	0.25
Biventricular failure (n, %)	4 (10%)	2 (20%)	2 (6.6%)	0.25
Right ventricular failure (n, %)	0 (0%)	0 (0%)	0 (0%)	-
SSc-HI				
Vasculopathy related-lesions (n, %)	15 (37.5%)	3 (30%)	12 (40%)	0.71
Fibrosis related-lesions (n, %)	8 (20%)	5 (50%)	3 (10%)	0.01
Myocardial involvement (n, %)	20 (50%)	7 (70%)	13 (43.3%)	0.27
Any SSc-HI (n, %)	22 (55%)	8 (80%)	14 (46.7%)	0.08

CMR: cardiac magnetic resonance; GLS: global longitudinal strain; LGE: late gadolinium enhancement; LV: left ventricle; MBF: myocardial blood flow; MPR: myocardial perfusion reserve; RV: right ventricle; SD: scleroderma; SSc: systemic sclerosis

Any SSc-HI was described in 22 (55%) SSc patients. Among them, vasculopathy-related lesions were described in 15 (37.5%) patients, of which 2 (5%) had cold-induced PD and 13 (38.2%) had an MPR < 1.0. Fibrosis-related lesions were detected in 8 (20%) patients, of whom 4 (10%) had LGE fibrotic involvement, 5 (12.5%) had an LV-GLS < 14%, and 4 (10%) had biventricular failure (Table 2).

The group of patients with the late SD pattern of NVC had a significantly higher indexed LV mass and a lower LV-GLS than patients with the non-late SD pattern did (60.7 ± 11.0 g versus 48.7 ± 9.7 g, *p* < 0.01; 14 ± 3% versus 17 ± 2%, *p* < 0.01, respectively). Moreover, the late SD group had a significantly higher frequency of moderate/severe pericardial effusion (*p* = 0.04) and a higher frequency of fibrosis-related lesions (*p* = 0.01), with more patients presenting with an LV-GLS < 14% (*p* = 0.01) and LGE (*p* < 0.01) than the results observed in the patients with the non-late SD pattern (Table 2).

Severe microangiopathy in NVC depicted by reduced capillary density was associated with different SSc-HI CMR abnormalities. As shown in Table 3, the mean number of capillary loops/mm was lower in patients with LGE (*p* = 0.01), an LV-GLS < 14% (*p* = 0.04), and biventricular failure (*p* = 0.03). There was a trend towards a lower number of capillary loops/mm in patients with an MPR < 1.0 (*p* = 0.06) compared to those without these abnormalities. Moreover, the avascular score was significantly higher in patients with an MPR < 1.0 (*p* = 0.04), LGE (*p* < 0.01) and an LV-GLS < 14% (*p* = 0.04). A significantly lower number of capillary loops/mm and higher avascular score were observed in patients with fibrosis-related lesions (3.7 ± 2.0 vs. 6.0 ± 2.0 loops/mm, *p* < 0.01 and 2.2 ± 0.9 vs. 1.1 ± 0.9, *p* = 0.01, respectively), myocardial involvement (4.4 ± 2.1 vs. 6.4 ± 1.9 loops/mm, *p* < 0.01 and 1.8 ± 0.9 vs. 0.9 ± 0.8, *p* < 0.01, respectively) and any SSc-HI (4.7 ± 2.2 vs. 6.4 ± 2.0 loops/mm, *p* = 0.01 and 1.7 ± 0.9 vs. 0.8 ± 0.8, *p* < 0.01, respectively) compared to those without these abnormalities (Table 3).

**Table 3:** Comparison of CMR findings and videocapillaroscopy (NVC) parameters

CMR parameters	NVC parameters (mean ± SD)	Present	Absent	*p* value
Cold-induced perfusion defects	Capillary loops/mm	5.8 ± 0.1	5.5 ± 2.2	0.92
	Avascular score	1.2 ± 0.1	1.3 ± 1.0	0.27
	Giant capillary	0.1 ± 0.1	0.4 ± 0.7	0.89
MPR < 1.0	Capillary loops/mm	4.8 ± 2.1	6.2 ± 2.1	0.06
	Avascular score	1.7 ± 1.0	1.0 ± 1.0	0.04
	Giant capillary	0.7 ± 0.9	0.3 ± 0.5	0.11
Moderate/severe pericardial effusion	Capillary loops/mm	4.4 ± 1.9	5.6 ± 2.2	0.30
	Avascular score	1.9 ± 0.7	1.3 ± 1.0	0.28
	Giant capillary	0.02 ± 0.0	0.5 ± 0.7	0.19
LGE	Capillary loops/mm	2.8 ± 1.0	5.8 ± 2.1	0.01
	Avascular score	2.6 ± 0.5	1.2 ± 0.9	< 0.01
	Giant capillary	0.2 ± 0.4	0.5 ± 0.7	0.55
LV-GLS < 14%	Capillary loops/mm	3.6 ± 2.3	5.8 ± 2.1	0.04
	Avascular score	2.2 ± 1.0	1.2 ± 0.9	0.03
	Giant capillary	0.2 ± 0.4	0.5 ± 0.7	0.40
Biventricular failure	Capillary loops/mm	3.3 ± 1.8	5.8 ± 2.1	0.03
	Avascular score	2.3 ± 0.8	1.2 ± 1.0	0.07
	Giant capillary	0.5 ± 0.5	0.4 ± 0.7	0.92
Vasculopathy related-lesions	Capillary loops/mm	4.9 ± 2.0	5.9 ± 2.2	0.19
	Avascular score	1.7 ± 0.8	1.1 ± 1.0	0.12
	Giant capillary	0.6 ± 0.9	0.3 ± 0.5	0.26
Fibrotic related-lesions	Capillary loops/mm	3.7 ± 2.0	6.0 ± 2.0	< 0.01
	Avascular score	2.2 ± 0.9	1.1 ± 0.9	0.01
	Giant capillary	0.3 ± 0.4	0.5 ± 0.7	0.75
Myocardial involvement	Capillary loops/mm	4.4 ± 2.1	6.4 ± 1.9	< 0.01
	Avascular score	1.8 ± 0.9	0.9 ± 0.8	< 0.01
	Giant capillary	0.5 ± 0.8	0.4 ± 0.6	0.55
Any SSc-HI	Capillary loops/mm	4.7 ± 2.2	6.4 ± 2.0	0.01
	Avascular score	1.7 ± 0.9	0.8 ± 0.8	< 0.01
	Giant capillary	0.5 ± 0.8	0.4 ± 0.6	0.70

CMR: cardiac magnetic resonance; GLS: global longitudinal strain; LGE: late gadolinium enhancement; LV: left ventricle; MPR: myocardial perfusion reserve

LV indexed mass was negatively correlated with the mean number of capillary loops/mm (*r* = -0.33, *p* = 0.03).

## Discussion

CMR is an accurate method for the evaluation of cardiac involvement in SSc patients [7, 8, 45]. To the best of our knowledge, for the first time, we evaluated associations between CMR and cold-induced vasoreactivity and peripheral microvascular abnormalities assessed by NVC in SSc patients.

The current study confirms the high frequency of CMR abnormalities in patients with SSc. The presence of SSc-HI on CMR was found in 55% of patients, which is in accordance with previous reports [12, 22, 46, 47]. SSc-HI changes related to the three distinct SSc pathogenic mechanisms of vasculopathy, inflammation, and fibrosis were observed in our study population. Indeed, abnormalities such as cold-induced perfusion abnormalities with perfusion defects or an MPR < 1.0, pericardial effusion, LGE, a reduced LV-GLS, and biventricular failure were found, highlighting the heterogeneity of cardiac manifestations in this population.

Concerning NVC, we found a higher frequency of SSc-HI changes on CMR in patients who had more severe peripheral microangiopathy, characterized by severe capillary loss with a lower mean number of capillary loops/mm or the presence of a late SD pattern. The late SD pattern was also associated with an increased frequency of moderate/severe pericardial effusion, indicating that inflammatory involvement of the pericardium may also be related to NVC abnormalities.

In addition to the important role of NVC in the diagnosis of SSc, increasing evidence suggests that peripheral microvascular abnormalities are associated with more severe disease manifestations [31, 35, 48]. One recent study revealed a correlation between reduced capillary density and inflammatory and fibrotic involvement of the heart, but unlike our study, only patients with PAH related to SSc (SSc-PAH) were evaluated and compared with those with idiopathic PAH [27]. SSc-PAH patients had lower capillary density than idiopathic PAH patients did, and reduced capillary density was correlated with fibrotic and inflammatory changes on T1 and T2 parametric mapping images. However, the population was enriched with moderate/severe SSc-PAH patients and had a high prevalence of the late SD pattern (90%). In contrast, in our study, we were also able to show fibrotic changes on CMR in a heterogeneous and more representative population of SSc patients, with a lower prevalence of the late SD pattern (25%) and no PAH.

To evaluate SSc-HI vasculopathy-related abnormalities via CMR and the vasodilatory response, we used a cold stress protocol during quantitative perfusion sequences adapted from the study by Galea et al. [12]. In that study, there was no difference in the mean MBF at rest or the MBF after cold stress between SSc patients and controls. However, compared with controls, SSc patients had a worse vasodilation response, with a lower mean percentage change in MBF after cold stress and a higher proportion of patients with delta MBF < 33.8%. Although our study had a higher temperature for cold stress (8–12 °C versus 0–4 °C), a similar pattern of vasodilatory response after cold stress was observed, with no increase in absolute MBF stress values and no significant difference in MPR or delta MBF < 33.8% between the late SD and non-late SD pattern groups. However, we also found a high proportion of patients with MBF < 33.8% (65%), reflecting an ineffective cardiac vasodilator response in SSc patients. Moreover, adopting a threshold of MPR < 1.0, which reflects worsened MBF after cold stress, we were able to find higher avascular scores and a trend towards a lower mean number of capillary loops/mm in patients with an MPR < 1.0. Thus, this is the first study that shows a relationship between cardiac perfusion changes assessed on CMR and peripheral structural changes in NVC, revealing an association between an ineffective cardiac vasodilatory response and worsening capillary changes.

Interestingly, higher avascular scores in NVC were found in those patients with CMR abnormalities related to vasculopathy (MPR < 1.0) and also to fibrotic changes. Thus, both classical pathogenic mechanisms of fibrosis and vasculopathy are present in SSc-HI and are associated with structural changes in peripheral microangiopathy. This finding may suggest that SSc-HI and peripheral microcirculation involvement share common pathogenic pathways, such as endothelial dysfunction associated with abnormal vasoreactivity with repeated episodes of microvasospasm that lead to ischaemic lesions associated with fibrosis and defective neoangiogenesis [1, 2]. Thus, the presence of more severe NVC abnormalities may support the indication of a CMR to better evaluate SSc-HI in clinical practice. Nonetheless, due to the low number of patients included in this study and the cross-sectional design, further studies are needed to confirm our results.

Furthermore, our study proposed the categorization of cardiac alterations by SSc-HI on CMR according to the main pathogenic mechanisms involved in SSc. Thus, cardiac abnormalities associated with cold-induced perfusion defects and an MPR < 1.0 were categorized as related to SSc-HI vasculopathy. LGE, a LV-GLS < 14%, and biventricular failure, were categorized as fibrosis-related to SSc-HI. Previous studies have shown an association between a reduced LV-GLS and LGE fibrotic changes [40–42]. In the study by Knight et al. [29], among the five cardiac phenotypes presented via CMR, the biventricular failure phenotype was associated with elevated native T1 values and a high proportion of LGE, both of which are indicative of fibrosis-related lesions [29].

In our study, SSc-HI related to vasculopathy was found in 37.5% of patients with SSc and related to fibrosis in 20% of SSc patients. However, T1 and T2 mapping sequences are considered the most sensitive techniques for detecting diffuse myocardial fibrosis and inflammation. As our CMR protocol did not assess inflammatory abnormalities, it was not possible to categorize inflammation-related lesions in SSc-HI patients. Moreover, fibrosis may be underestimated, and future studies studies incorporating T1 and T2 mapping are needed to better define the relative contributions of microvascular dysfunction, inflammation, and diffuse fibrosis in SSc-HI.

With respect to myocardial fibrosis assessed by LGE, we found a lower prevalence in our study (10%) than those presented in other studies, which varied from 15 to 83% depending on the SSc population [15, 47]. Nevertheless, other SSc-HI CMR findings related to myocardial fibrosis, such as an LV-GLS and biventricular failure according to the volumetric and functional classification of Knight et al. [29] were also associated with capillary loss and a late SD pattern.

Our study has several limitations, including the small sample size, which requires further validation in studies of larger populations. Second, to represent a more heterogeneous population of SSc patients, it was decided not to enrich the study population with SSc patients with known risk factors associated with SSc-HI; therefore, a low prevalence of some SSc-HI CMR findings, such as LGE or NVC with an SD late pattern, was present, which may have impacted further analysis. Parametric T1 and T2 mapping sequences, which are more sensitive for evaluating diffuse myocardial fibrosis and myocardial inflammation, were unavailable. Furthermore, the cross-sectional design of the study did not allow for prospective analyses of NVC risk factors associated with worse CMR findings or clinical outcomes.

## Conclusions

SSc-HI CMR abnormalities, which are mediated by different pathogenic mechanisms related to vasculopathy, inflammation, and fibrosis, are frequently observed in SSc patients. The cardiac abnormalities observed on CMR are associated with several parameters of severity in capillaroscopy, such as a lower mean number of capillary loops/mm, a higher avascular score, and the presence of a late SD pattern. The structural abnormalities of the peripheral microcirculation observed in NVC may distantly mirror the cardiac abnormalities associated with SSc-HI assessed via CMR, thus suggesting a possible shared pathogenic pathway in the peripheral microcirculation and heart. Further studies, with a larger number of patients are needed to confirm the association between SSc-HI characterized by CMR and capillaroscopy abnormalities.

## Supplementary Information

Below is the link to the electronic supplementary material.


Supplementary Material 1


## Data Availability

The data that support this study are available on a reasonable request to the corresponding author.

## Abbreviations

AHA: American Heart Association
AIF: arterial input fraction
CMR: cardiac magnetic resonance
LV: left ventricular
LGE: late gadolinium enhancement
LV-GLS: left ventricular global longitudinal strain
MPR: myocardial perfusion reserve
MBF: myocardial blood flow
NVC: videocapillaroscopy
PAH: pulmonary arterial hypertension
PD: perfusion defects
RV: right ventricular
SD: scleroderma
SSc: systemic sclerosis
SSc-HI: heart involvement associated with systemic sclerosis
