# Supplementary material for: Primary heart involvement assessed by cardiac magnetic resonance is associated with capillaroscopy abnormalities in systemic sclerosis
Source: Adv Rheumatol. 2026 Jun 19;66:51. doi: 10.1186/s42358-026-00549-9 (PMC13528102; doi:10.1186/s42358-026-00549-9)
Supplement: Supplementary file 1 [file 42358_2026_549_MOESM1_ESM.docx]

Supplementary material

**Supplementary Data 1.**

1. **CINE-MRI**

After a multiplanar localization sequence to define the cardiac axes, a steady state free precession (SSFP) CINE-MRI sequence was performed with breath-hold during expiration to assess the morphology and function in the short and long axes of the heart (two chambers, left ventricular (LV) outflow tract, and four chambers).

2. **Perfusion Imaging**

Initially, a perfusion mask sequence was performed at rest. This sequence determined the 3 slices of the LV short axis (basal, middle, and apical) used for the arterial input fraction (AIF) and perfusion sequences at rest and after cold stress.

Subsequently, sequence of AIF at was started. First, contrast was injected, followed by a bolus of 20mL of 0.9% saline solution controlled by an injection pump. In the 3 pre-determined LV sections, images of the passage of contrast through the LV until its washout were acquired. Then, the same protocol was repeated for the rest perfusion sequence. The dose of contrast for the AIF sequence was 1mL of Gadovist, and the perfusion at rest sequence was adjusted according to the patient's weight (0.15mL/Kg). There was a 3-minute pause between the acquisition of the two sequences to allow better contrast washout.

Cold stress was then applied by immersing the hand and wrist in a container of water at a temperature of 10° ± 2 °C for 3 minutes, and then the stress AIF and stress perfusion sequences were acquired using the same protocol as the images at rest.

3. **Late gadolinium enhancement sequence**

The last stage of image acquisition consisted of the high-resolution phase-sensitive inversion recovery (PSIR) delayed enhancement sequence. This sequence was started 5 minutes after the injection of the contrast used at stress perfusion with cold stress.

4. **Post-processing**
   a. **Morphology and function**

The short-axis CINE-MRI images were used for morphological and functional analysis. Volume, mass, and function measurements were calculated using the CVI42 software. The papillary muscles were included as part of the heart cavity. The indexed measurements were calculated according to the body surface area derived from weight and height.

b. **LV and RV Strain**

LV and RV strain were calculated from the CINE-MRI sequence images. When assessing LV strain, the endocardial and epicardial contours of all LV long-axis sections were marked. The marking of the contours was semi-automated using the CVI42 software with subsequent manual correction of the contours. The same software then calculated the LV strain using the feature tracking method, with maximum diastole as the reference point in the cardiac cycle for calculating global LV longitudinal strain. The final GLS-LV result was provided in percentage values. The RV strain was also analyzed from the CINE-MRI sequence images. To assess RV strain, the endocardial contour was marked semi-automatically using the CVI42 software, with subsequent manual correction of the contours. The epicardial contour was marked manually. The software then calculated the RV strain using the feature tracking method, with maximum diastole as the reference point in the cardiac cycle for calculating RV strain. The RV-GLS result was recorded in percentage values.

c. **Cold-induced perfusion defects**

Perfusion defects were defined as the presence of myocardial areas of contrast hypointensity during contrast passage.

d. **Quantitative perfusion**

Initially, the CVI42 software semi-automatically marked the endocardial and epicardial contours of the 3 predetermined LV short-axis sections. When necessary, the markings on the LV short-axis sections were corrected manually. The software then calculated myocardial blood flow (MBF) at rest and stress. Both MBF results were displayed on polar maps with values for each of the 16 out of 17 cardiac segments in the AHA cardiac segmentation model (excluding the cardiac apex). The final MBF value at rest or stress was the mean MBF value of the 16 cardiac segments. Subsequently, the myocardial perfusion reserve (MPR), the ratio between stress MBF and rest MBF, was calculated.

e. **Myocardial fibrosis**

Myocardial fibrosis was assessed by hypersignal in late gadolinium enhancement sequences where a non-ischemic epimesocardial or mesocardial pattern was observed.

f. **Volumetric and functional classification**

The values of indexed RV end-diastolic volume, indexed RV end-systolic volume, indexed LV end-systolic volume, indexed LV end-diastolic volume, and LV ejection fraction were used to classify SSc patients into 5 volumetric and functional phenotypes: normal function and a small cavity, normal function and a medium cavity, normal function and a large cavity, biventricular failure, and right ventricular failure, according to the flowchart proposed in the study by Knight et al.

g. **Pericardial involvement**

Pericardial involvement was analyzed by visually identifying the presence of pericarditis and/or pericardial effusion. In the presence of pericardial effusion, it was measured and classified as laminar or mild pericardial effusion (up to 10 mm), moderate pericardial effusion (between 11-20 mm), or significant pericardial effusion (over 20 mm).

**Supplementary Table S1.** Cardiac magnetic resonance parameters for image acquisition

| **Parameters** | **CINE-MRI** | **Quantitative perfusion** | **Late gadolinium enhancement** |
| --- | --- | --- | --- |
| TR (ms) | 49.5 | 173.4 | 600 |
| TE (ms) | 1.15 | 1.06 | 1.03 |
| Phases (n) | 25 | 60 | 11 |
| Slice thickness (mm) | 8.0 | 10.0 | 8.0 |
| Inversion time (ms) | 0.0 | 100 | 300-400 |
| Image size (mm) | 232 x 256 | 144 x 192 | 192 x 192 |
| Flip angle (^o^) | 60 | 12 | 40 |
| Bandwidth (Hz/Pixel) | 930 | 685 | 1532 |

TE: echo time; TR: repetition time
